# Decoding the underlying mechanisms of Di-Tan-Decoction in treating intracerebral hemorrhage based on network pharmacology

**DOI:** 10.1186/s12906-022-03831-7

**Published:** 2023-02-10

**Authors:** Zheng Zhen, Dao-jin Xue, Yu-peng Chen, Jia-hui Li, Yao Gao, You-bi Shen, Zi-zhuang Peng, Nan Zhang, Ke-xin Wang, Dao-gang Guan, Tao Huang

**Affiliations:** 1 The Second Affiliated Hospital of Guangzhou University of Chinese Medicine, Guangzhou, China; 2 Department of Biochemistry and Molecular Biology, School of Basic Medical Sciences, Southern Medical University, Guangzhou, China; 3 Guangdong Provincial Key Laboratory of Single Cell Technology and Application, Guangzhou, Guangdong Province China; 4 Department of Psychiatry, First Hospital/First Clinical Medical College of Shanxi Medical University, Taiyuan, 030001 China; 5 Neurosurgery Center, Guangdong Provincial Key Laboratory On Brain Function Repair and Regeneration, Department of Cerebrovascular Surgery, Engineering Technology Research Center of Education Ministry of China On Diagnosis and Treatment of Cerebrovascular Disease, Zhujiang Hospital, Southern Medical University, Guangzhou, 510280 Guangdong China; 6 Department of Bioinformatics, School of Basic Medical Sciences, Southern Medical University, Guangzhou, China

**Keywords:** Traditional chinese medicine (TCM), Intracerebral hemorrhage (ICH), Important gene network motif, Mechanism, System pharmacology

## Abstract

**Background:**

Chinese medicine usually acts as "multi-ingredients, multi-targets and multi-pathways" on complex diseases, and these action modes reflect the coordination and integrity of the treatment process with traditional Chinese medicine (TCM). System pharmacology is developed based on the cross-disciplines of directional pharmacology, system biology, and mathematics, has the characteristics of integrity and synergy in the treatment process of TCM. Therefore, it is suitable for analyzing the key ingredients and mechanisms of TCM in treating complex diseases. Intracerebral Hemorrhage (ICH) is one of the leading causes of death in China, with the characteristics of high mortality and disability rate. Bring a significant burden on people and society. An increasing number of studies have shown that Chinese medicine prescriptions have good advantages in the treatment of ICH, and Ditan Decoction (DTT) is one of the commonly used prescriptions in the treatment of ICH. Modern pharmacological studies have shown that DTT may play a therapeutic role in treating ICH by inhibiting brain inflammation, abnormal oxidative stress reaction and reducing neurological damage, but the specific key ingredients and mechanism are still unclear.

**Methods:**

To solve this problem, we established PPI network based on the latest pathogenic gene data of ICH, and CT network based on ingredient and target data of DTT. Subsequently, we established optimization space based on PPI network and CT network, and constructed a new model for node importance calculation, and proposed a calculation method for PES score, thus calculating the functional core ingredients group (FCIG). These core functional groups may represent DTT therapy for ICH.

**Results:**

Based on the strategy, 44 ingredients were predicted as FCIG, results showed that 80.44% of the FCIG targets enriched pathways were coincided with the enriched pathways of pathogenic genes. Both the literature and molecular docking results confirm the therapeutic effect of FCIG on ICH via targeting MAPK signaling pathway and PI3K-Akt signaling pathway.

**Conclusions:**

The FCIG obtained by our network pharmacology method can represent the effect of DTT in treating ICH. These results confirmed that our strategy of active ingredient group optimization and the mechanism inference could provide methodological reference for optimization and secondary development of TCM.

**Supplementary Information:**

The online version contains supplementary material available at 10.1186/s12906-022-03831-7.

## Introduction

Hypertensive intracerebral hemorrhage (ICH) is one of the main causes of death and disability among Chinese residents. According to the statistics, the mortality rate is as high as 35%-52%, and about 80% of the surviving patients still have disabilities within half a year [1], which brings great burden to individuals and society. ICH mostly occurs in the area of perforator arteries. Under the influence of long-term hypertension, these perforating arteries will have a series of pathological changes, such as "lipid hyaline degeneration", hyperplasia of subendothelial fibroblasts, deposition of macrophages, and replacement of collagen-rich medial smooth muscle cells. These changes result in decreased vascular compliance and lumen stenosis, which is prone to cerebral hemorrhage when blood pressure fluctuates greatly. Modern medicine mainly treats ICH by controlling blood pressure, clearing hematoma and preventing rebleeding. Some basic studies in recent years have revealed some key mechanisms for treating cerebral hemorrhage. Isoliquiritigenin reduces early brain injury following experimental intracerebral hemorrhage by suppressing ROS- and/or NF-B-mediated NLRP3 inflammasome activation via the Nrf2 antioxidant pathway [2]. Following ICH, the Striatal P2X7R and NLRP3 inflammasomes were activated. P2X7R gene silencing inhibited NLRP3 inflammasome activation and interleukin (IL)-1/IL-18 release, significantly alleviating brain edema and neurological deficits [3].

In recent years, many experimental studies have been carried out on ICH. Statins can improve neurological outcome and promote neurovascular recovery after ICH [4]. Mesenchymal stromal cells-derived exosomes effectively improve functional recovery after ICH [5]. However, due to the limited treatments and effects, there are still great challenges to the problems such as the disappearance of brain edema, rebleeding after treatment, and recovery after nerve function injury. Increasing evidence confirms that the prescriptions of TCM have been widely used in the treatment of ICH, Ling et al. investigate that Tongfu Xingshen Decoction can improve clinical symptoms of patients and promote the recovery of neurological function by reducing serum S100β, IL-6 and MMP-9 levels [6]. Guo et al. reported that Angong Niuhuang Pill can alleviate the brain damage caused by ICH from diuresis and dehydration, reducing intracranial pressure and protecting central nervous system [7]. Zhou et al. confirmed that DTT can promote the recovery of cognitive function and improve the clinical efficacy in patients with ICH [8]. Among these prescriptions, DTT is one of the most high frequency used in clinic.

The DTT prescription contains 9 herbs: *Glycyrrhiza uralensis* Fisch. ex DC.(1.5 g), *Zingiber officinale Roscoe* (1.5 g), *Citrus reticulata* Blanco (2.1 g), *Arisaema heterophyllum* Blume (2.5 g). *Panax ginseng C.A.*Mey*.*(3 g), *Pinellia ternata (Thunb.)* Makino(3 g), *Poria cocos (Schw.)* Wolf*.*(3 g), *Citrus* *acida* Pers (2.5 g), *Bambusa tuldoides* Munro (2.1 g).

The prescriptions of TCM have multiple ingredients, multiple targets and multiple pathways, which are necessary factors for treating many complex diseases. As a new subject, network pharmacology is beneficial to identify the effective ingredients and explore the mechanisms of TCM in modern pharmacology research. It is helpful to explain the "combination-effect relationship" and compatibility rule of TCM at a system level. The effect of TCM on cells and organisms is a complex biological network, which is inline with the "integrity" characteristics of systems pharmacology. Thus, system pharmacology is suitable for studying the underlying rules of TCM prescription.

At present, several formula optimization methods have been proposed. Most of these methods take the structural similarity of ingredients as the most important feature but ignore the linkage of pathogenic genes and drug targets. In this study, systems pharmacology was used to detect FCIG and clarify the mechanisms of DTT in treating ICH. We present a reverse optimization model based on the association between disease genes and ingredient targets is proposed, which provides space for optimization based on effective proteins. This method can well determine the optimization space of the target. Second, reverse searching related ingredients based on the optimized space provided by effective protein. The results showed that the enrichment functional pathway of effective proteins could cover 96% of the enrichment functional pathway of disease genes.

## Materials and methods

### Construct weighted gene regulatory network of ICH

PPI data derived from Dip [9], HPRD [10], BioGRID [11], STRING [12], Reactome [13], Mint [14] and Intact [15] were used to construct comprehensive protein–protein interaction network (Supplementary Table 1). ICH related genes were extracted from GeneCards. The genes with “Relevance Scores” higher than average score were kept as high pathogenic gene. These pathogenic genes are aligned into the PPI network to figure out the weighted gene regulatory network of ICH. The Cytoscape software was utilized to visualize the network.

### Collect chemical ingredients of DTT

Ingredients of DTT were extracted from three herbal medicine databases: TCMID and TCMSP. For all ingredients, the OpenBabel toolkit (version 2.4.1) was employed to convert the format of structure to canonical SMILES. Subsequently, the chemical properties, such as molecular weight (MW), DL (drug-likeness), Caco-2 permeability (Caco-2) and oral bioavailability (OB) were retrieved from TCMSP.

### Select potential active ingredients of DTT based on ICH models

Three published ADME-related modules, OB, Caco-2 and DL, were used to select the bio-active ingredients. OB (%F) refers to the relative amount and rate of absorption of drugs into circulation after oral administration. High oral bio-availability is one of the key indicators to determine the therapeutic properties of drugs. Selecting suitable ingredients with OB ≥ 30% as potential active ingredients for next step analysis. Caco-2 cell model is a human clonal colon adenocarcinoma cell, which has similar structure and function as intestinal epithelial cells, and it contains an enzyme system related to the brush border epithelium of the small intestine, which can be used to simulate the intestinal transport of drugs in vivo. The transport rate (nm/s) of ingredients in Caco-2 cells represents the permeability of intestinal epithelium. Ingredients with Caco-2 value less than -0.4 have poor permeability in intestinal epithelial cells, so we choose ingredients with Caco-2 > -0.4 as candidate ingredients. DL is used to evaluate the drug-like properties of expected compounds, which is helpful to optimize pharmacokinetics and drug characteristics, such as solubility and chemical stability. In this study, the drug-like scores of the ingredients were more than 0.18 as the selection criteria [16].

After screening by ADME, some ingredients that do not meet the three screening criteria are also selected, because experiments have proved that they have high content and high biological activity. These high-content and high-activity ingredients were merged with ADMET screened ingredients for subsequent analysis.

### Predicting targets of active ingredients

To obtain the targets of active ingredients in DTT, the widely used prediction tools, HitPick, Swiss Target Prediction and Similarity Ensemble Approach (SEA) [8] and Swiss Target Prediction were used to predict the targets. All chemical structures were converted to SMILE format.

### Node importance calculation method

For calculating the importance of each node in the network, we constructed a Node importance calculation method (BCD), in which, *m* represent the number of nodes in the network; *h*, *i* and *j* represent nodes in the network, *V* represent the unit of all nodes in the network, ${\upsigma }_{hi}$ represents the number of the shortest path between nodes *h* and *i*; ${\upsigma }_{hi \left(j\right)}$ is the number of the shortest path passing through node *j*.${\mathrm{T}}_{h}$ is the number of edges directly connected to a node *h*. N stands for natural number. The method can be described as follows:

$${\mathrm{BD}}_{h} = {\sqrt{{\mathrm{ T}}_{h} \times ( \sum_{h \ne i \ne j \in V}\frac{{\upsigma }_{hi(j)}}{{\upsigma }_{hi}} )}}$$

There are m nodes in the network. Combining degree with the center degree of betweenness, BD can effectively reflect the direct relationship between nodes and neighboring nodes, as well as the control function of neighboring nodes in the whole network [17–19].

After being quantized, BD was sorted from small to large, and was represented by a new variable Y.

$$\mathrm{Y}=\left[{\mathrm{Y}}_{1}, {\mathrm{Y}}_{2}, {\mathrm{Y}}_{3}, \dots , {\mathrm{Y}}_{\mathrm{m}}\right]= \left[{\mathrm{BD}}_{\mathrm{x}}, {\mathrm{BD}}_{\mathrm{x}+1}, {\mathrm{BD}}_{\mathrm{x}+2}, \dots , {\mathrm{BD}}_{\mathrm{x}+\mathrm{n}}\right],\mathrm{ x}\in \left[1,\mathrm{ m}\right]\mathrm{ and x}+\mathrm{n}=\mathrm{m}$$

The new variable R represented the nodes in the network. Each R responds to its unique Y.

$$\mathrm{R }\in \left\{{\mathrm{R}}_{1}, {\mathrm{R}}_{2}, {\mathrm{R}}_{3}, \dots , {\mathrm{R}}_{\mathrm{m}}\right\} \rightleftharpoons \left[{\mathrm{Y}}_{1}, {\mathrm{Y}}_{2}, {\mathrm{Y}}_{3}, \dots , {\mathrm{Y}}_{\mathrm{m}}\right]$$

BCD represented the important nodes selected form all nodes in the network. N represented natural number.

$$\mathrm{BCD}\in \left\{\begin{array}{c}\left\{{\mathrm{R}}_{\mathrm{r}} , {\dots ,{\mathrm{R}}_{(\mathrm{m}-2)} , {\mathrm{R}}_{(\mathrm{m}-1)} ,\mathrm{ R}}_{\mathrm{m}}\right\} \rightleftharpoons \left[{\mathrm{Y}}_{\left(\mathrm{m}+1\right)/2} , {\dots ,{\mathrm{Y}}_{(\mathrm{m}-2)} , {\mathrm{Y}}_{(\mathrm{m}-1)} ,\mathrm{ Y}}_{\mathrm{m}}\right], m=2p+1 and p\in N\\ \\ \\ \left\{{\mathrm{R}}_{\mathrm{r}} ,\dots , {\mathrm{R}}_{(\mathrm{m}-2)} , {\mathrm{R}}_{(\mathrm{m}-1)} , {\mathrm{R}}_{\mathrm{m}}\right\} \rightleftharpoons \left[\frac{({\mathrm{Y}}_{\mathrm{m}/2}+ {\mathrm{Y}}_{(\mathrm{m}+2)/2})}{2} ,\dots , {\mathrm{Y}}_{(\mathrm{m}-2)} , {\mathrm{Y}}_{(\mathrm{m}-1)} , {\mathrm{Y}}_{\mathrm{m}}\right], m=2p and p\in N\end{array}\right.$$

We collected all ingredients of DTT from databases and literature. All ingredients of DTT were used to screen out potential active ingredients by using previous reported ADME models. Three public online tools were employed to identify the targets of the active ingredients. The active ingredients and their targets were used to construct CT network. The pathogenic genes were mapped to a comprehensive protein–protein interaction network to obtain a pathogenic gene regulation network. CT network and pathogenic gene regulation network were integrated and then used to find potential effect space (PES) by using novel node importance calculation model. Finally, the contribution index (CI) model was employed to optimize the FCIG in the PES, and the key functional ingredients were obtained, and the molecular mechanism of DTT on ICH was expounded.

### Gene ontology and pathway analysis

Function enrichment analysis is the basic way to understand gene function. Here, the clusterProfiler of R package were performed to conduct pathway and GO term enrichment analysis. The p value less than 0.05 were considered to be functionally related pathways and GO terms.

### Experimental validation

#### Materials

Jiangsu Yongjian Biotech Co., Ltd provided 6-shogaol, 6-singerol, kaempferol, and sitostrol (98% purity by HPLC) (Chengdu, China). Gibco supplied fetal bovine serum (FBS) and Dulbecco's modified Eagle's medium (DMEM) (Grand Island, USA). CHI SCIENTIFIC provided the mouse hippocampal HT-22 cells (Shanghai, China). Mitsubishi Gas Chemical Company, Inc. provided the hypoxic bags (Japanese). Dojindo Laboratories supplied the Cell Counting Kit-8 (CCK-8) (Japanese).

#### Cell Culture and oxygen–glucose deprivation (ogd) treatment

The cells were cultured in DMEM with 10% FBS, and incubated at 37 °C under 5% CO_2_. Hypoxic bags were used to perform OGD model according to the methods in the literature [20].

#### Cell viability assay

HT22 cells (6 × 10^4^ cells/well) were seeded in 96-well plates, and treated with different concentrations of 6-Shogaol, 6-Singerol, kaempferol, and sitostrol (40, 80, 120, 160 and 200 μM). CCK8 was superinduced to 96-well plate for 4 h. The plate reader was utilized to detect the absorbance at 450 nm.

#### Statistical analysis

The data were all expressed as mean SEM. For multiple comparisons, one-way ANOVA was used, and the Student's t test was used to compare the significance of differences between two groups. If the p-value was less than 0.05, the results were considered statistically significant.

## Results

**Fig. 1:**
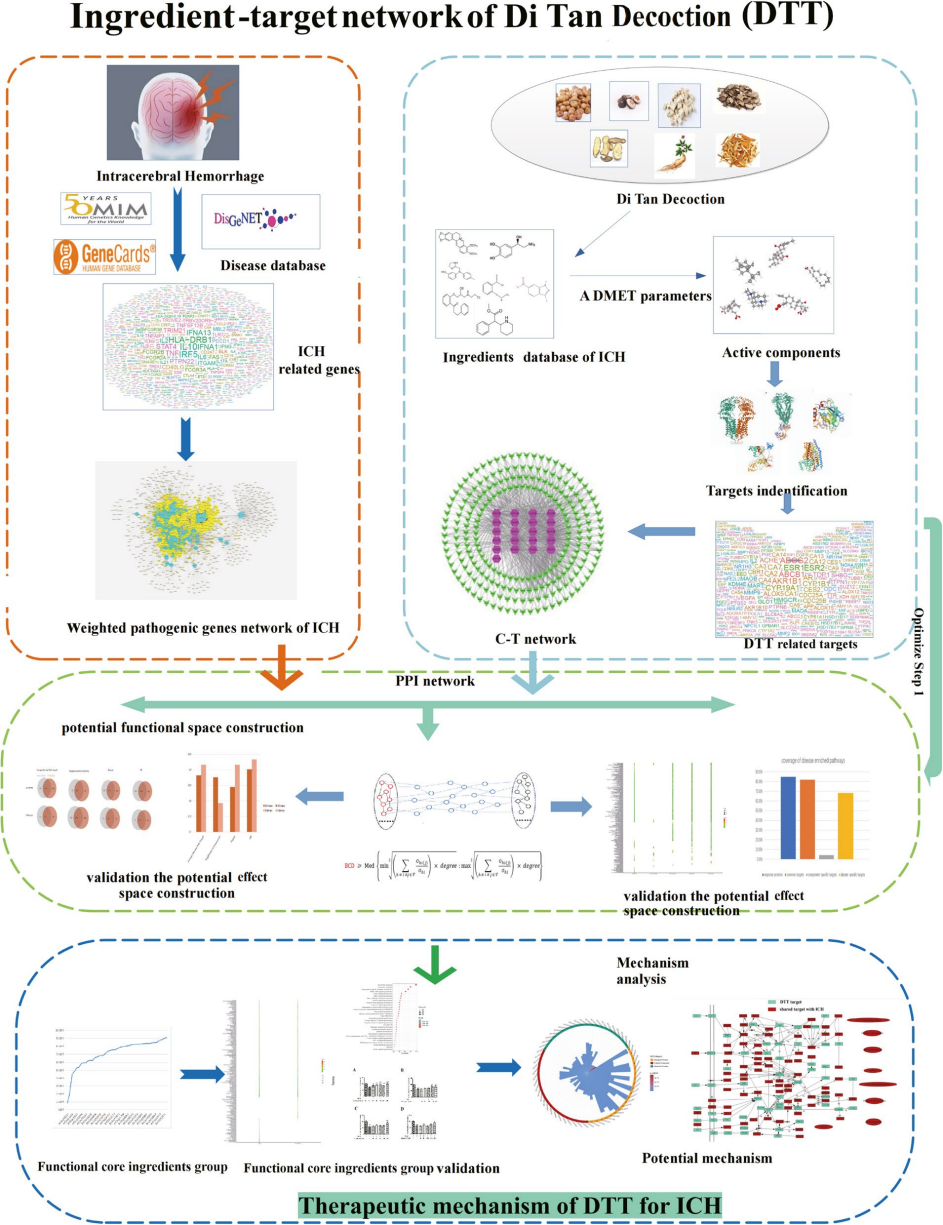
The work scheme of system pharmacology approach

In this study, we designed a new system pharmacology module, which was used to detect the key active ingredients and clarify the therapeutic mechanism of DTT in treating ICH. The work flow is illustrated in (Fig. 1) and described as follows: Firstly, all the effective ingredients of DTT were collected from databases and literature. The potential active ingredients were selected from DTT and predicted by three published prediction tools. Then, the weighted gene regulatory network and the active ingredient target network are used to construct the PES to determine the effective protein. Selecting key active ingredients based on CI module by using effective proteins. Finally, the molecular mechanism of DTT in treating ICH was deduced from the FCIG.

### Chemical analysis

Chemical identification is the critical step to clarify the material basis and action mechanism of compound prescription. In this study, the information of specific chemical ingredients of DTT Chinese herbal medicines was obtained from the literature (Table 1). The results showed that the chemical ingredients of herbs and the content of identified ingredients provided experimental auxiliary chemical space for searching active ingredients. The chemical constituent analysis serves as a reference for the screening of active constituents in DTT.

**Table 1:** The information on chemical analysis of the herbs from the literature in DTT

Herb	Method	Component	Concentration	Ref
*Glycyrrhiza uralensis* Fisch. ex DC	HPLC	Glycyrrhizin	98.01 mg/g	Chen et al., 2009 [21]
		Liquiritin	102.63 mg/g	
		Isoliquritigenin	98.18 mg/g	
*Zingiber officinale* Roscoe	HPLC	6-Gingerol	16.62 mg/g	Zhang et al., 2009 [22]
		6-Shogaol	4.92 mg/g	
*Citrus reticulata* Blanco	HPLC	Naringin	24.87 mg/g	Liu et al., 2013 [23]
		Hesperidin	2.19 mg/g	
*Arisaema heterophyllum* Blume	HPLC	Guanosine	0.436 mg/g	Zhang et al., 2020 [24]
		Adenosine	0.642 mg/g	
		Chafertoside	0.618 mg/g	
		Isochafertoside	0.517 mg/g	
*Panax ginseng C.A*.Mey	HPLC–MS	Rg1	0.205 mg/g	Shang et al., 2018 [25]
		Re	0.175 mg/g	
		Rf	0.050 mg/g	
		Rb1	0.112 mg/g	
		Rb2	0.033 mg/g	
		Rd	0.016 mg/g	
*Pinellia ternata* (Thunb.) Makino	RP-HPLC	Uridine	0.224 mg·g	Chen et al., 2013 [26]
		guanosine	0.337 mg·g	
		adenosine	0.084 mg·g	
*Poria cocos* (Schw.) Wolf	HPLC	Dehydrotumonic acid	0.34 mg/g	Peng et al., 2017 [27]
		dehydrofulic acid	0.29 mg/g	
		poriatic acid	0.72 mg/g	
		melinolic acid	0.15 mg/g	
*Citrus acida* Pers	HPLC	Naringenin	1.91 mg/g	Zhan et al., 2015 [28]
		Hesperetin	1.37 mg/g	
		Marmin	1.52 mg/g	
		6’,7’-Dihydroxy bergamot	2.96 mg/g	
		Citronella	2.90 mg/g	
		Orange peel	2.18 mg/g	
*Bambusa tuldoides* Munro	HPLC	Flavonoids	0.81 mg/mL	Li et al., 2017 [29]

### Select potential active ingredients

Although each TCM compound contains many ingredients, only a few ingredients have satisfactory pharmacodynamic and pharmacokinetic properties. In the present work, three ADME-related models, including OB, Caco-2 and DL, are used to screen active ingredients. After ADME screening, some ingredients not pass the criterion of three ADME-related models were kept as active ingredients due to their high content and high bio-activity. Finally, 181 active ingredients were captured from 939 active ingredients (Table 2).

**Table 2:** Components in DTT for further analysis after AD ME screening

Herb	MOL_ID	molecule_name	ob	caco2	drug-likeness
Fuling	MOL000273	(2R)-2-[(3S,5R,10S,13R,14R,16R,17R)-3,16-dihydroxy-4,4,10,13,14-pentamethyl-2,3,5,6,12,15,16,17-octahydro-1H-cyclopenta[a]phenanthren-17-yl]-6-methylhept-5-enoic acid	30.93	0.01	0.81
Fuling	MOL000275	trametenolic acid	38.71	0.52	0.80
Fuling	MOL000276	7,9(11)-dehydropachymic acid	35.11	0.03	0.81
Fuling	MOL000279	Cerevisterol	37.96	0.28	0.77
Fuling	MOL000280	(2R)-2-[(3S,5R,10S,13R,14R,16R,17R)-3,16-dihydroxy-4,4,10,13,14-pentamethyl-2,3,5,6,12,15,16,17-octahydro-1H-cyclopenta[a]phenanthren-17-yl]-5-isopropyl-hex-5-enoic acid	31.07	0.05	0.82
Fuling	MOL000282	ergosta-7,22E-dien-3beta-ol	43.51	1.32	0.72
Fuling	MOL000283	Ergosterol peroxide	40.36	0.84	0.81
Fuling	MOL000285	(2R)-2-[(5R,10S,13R,14R,16R,17R)-16-hydroxy-3-keto-4,4,10,13,14-pentamethyl-1,2,5,6,12,15,16,17-octahydrocyclopenta[a]phenanthren-17-yl]-5-isopropyl-hex-5-enoic acid	38.26	0.12	0.82
Fuling	MOL000287	3beta-Hydroxy-24-methylene-8-lanostene-21-oic acid	38.70	0.61	0.81
Fuling	MOL000289	pachymic acid	33.63	0.10	0.81
Fuling	MOL000290	Poricoic acid A	30.61	-0.14	0.76
Fuling	MOL000291	Poricoic acid B	30.52	-0.08	0.75
Fuling	MOL000292	poricoic acid C	38.15	0.32	0.75
Fuling	MOL000296	hederagenin	36.91	1.32	0.75
Fuling	MOL000300	dehydroeburicoic acid	44.17	0.38	0.83
Renshen	MOL000358	beta-sitosterol	36.91	1.32	0.75
Renshen	MOL000422	kaempferol	41.88	0.26	0.24
Renshen	MOL000449	Stigmasterol	43.83	1.44	0.76
Renshen	MOL000749	Linoleic	41.90	1.23	0.14
Renshen	MOL000787	Fumarine	59.26	0.56	0.83
Renshen	MOL001641	METHYL LINOLEATE	41.93	1.44	0.17
Renshen	MOL002879	Diop	43.59	0.79	0.39
Renshen	MOL003648	Inermin	65.83	0.91	0.54
Renshen	MOL004492	Chrysanthemaxanthin	38.72	0.51	0.58
Renshen	MOL005308	Aposiopolamine	66.65	0.66	0.22
Renshen	MOL005314	Celabenzine	101.88	0.77	0.49
Renshen	MOL005317	Deoxyharringtonine	39.27	0.19	0.81
Renshen	MOL005320	arachidonate	45.57	1.27	0.20
Renshen	MOL005321	Frutinone A	65.90	0.89	0.34
Renshen	MOL005348	Ginsenoside-Rh4_qt	31.11	0.50	0.78
Renshen	MOL005356	Girinimbin	61.22	1.72	0.31
Renshen	MOL005357	Gomisin B	31.99	0.60	0.83
Renshen	MOL005360	malkangunin	57.71	0.22	0.63
Renshen	MOL005366	Malvic acid	30.99	1.22	0.15
Renshen	MOL005376	Panaxadiol	33.09	0.82	0.79
Renshen	MOL005384	suchilactone	57.52	0.82	0.56
Renshen	MOL005396	cis-Widdrol alpha-epoxide	69.04	1.07	0.15
Renshen	MOL005399	alexandrin_qt	36.91	1.30	0.75
Renshen	MOL005401	ginsenoside Rg5_qt	39.56	0.88	0.79
Juhong	MOL000358	beta-sitosterol	36.91	1.32	0.75
Juhong	MOL001040	(2R)-5,7-dihydroxy-2-(4-hydroxyphenyl)chroman-4-one	42.36	0.38	0.21
Juhong	MOL001798	neohesperidin_qt	71.17	0.26	0.27
Juhong	MOL001942	isoimperatorin	45.46	0.97	0.23
Juhong	MOL004328	naringenin	59.29	0.28	0.21
Juhong	MOL005849	didymin	38.55	0.60	0.24
Juhong	MOL013331	Isomeranzin	42.78	0.69	0.15
Juhong	MOL013332	Pranferol	38.29	0.54	0.25
Juhong	MOL013352	Obacunone	43.29	0.01	0.77
Tiannanxing	MOL000131	EIC	41.90	1.16	0.14
Tiannanxing	MOL000358	beta-sitosterol	36.91	1.32	0.75
Tiannanxing	MOL000359	sitosterol	36.91	1.32	0.75
Tiannanxing	MOL000432	linolenic acid	45.01	1.21	0.15
Tiannanxing	MOL000449	Stigmasterol	43.83	1.44	0.76
Tiannanxing	MOL000953	CLR	37.87	1.43	0.68
Tiannanxing	MOL001510	24-epicampesterol	37.58	1.43	0.71
Tiannanxing	MOL001641	METHYL LINOLEATE	41.93	1.44	0.17
Tiannanxing	MOL002203	Exceparl M-OL	31.90	1.39	0.16
Tiannanxing	MOL010925	Isooleic acid	33.13	1.15	0.14
Tiannanxing	MOL013131	12,15-Octadecadienoic acid, methyl ester	41.93	1.46	0.17
Tiannanxing	MOL013144	Methyl (6E,9E)-6,9-octadecadienoate	41.93	1.44	0.17
Tiannanxing	MOL013145	Methyl-7,10-octadecadienoate	41.93	1.43	0.17
Tiannanxing	MOL013146	8,11,14-Docosatrienoic acid, methyl ester	43.23	1.53	0.30
Tiannanxing	MOL013156	[(2R)-2-[[[(2R)-2-(benzoylamino)-3-phenylpropanoyl]amino]methyl]-3-phenylpropyl] acetate	38.88	0.35	0.56
Banxia	MOL000131	EIC	41.90	1.16	0.14
Banxia	MOL000358	beta-sitosterol	36.91	1.32	0.75
Banxia	MOL000432	linolenic acid	45.01	1.21	0.15
Banxia	MOL000449	Stigmasterol	43.83	1.44	0.76
Banxia	MOL000519	coniferin	31.11	0.42	0.32
Banxia	MOL000675	oleic acid	33.13	1.17	0.14
Banxia	MOL001755	24-Ethylcholest-4-en-3-one	36.08	1.46	0.76
Banxia	MOL002495	6-shogaol	31.00	1.07	0.14
Banxia	MOL002670	Cavidine	35.64	1.08	0.81
Banxia	MOL002714	baicalein	33.52	0.63	0.21
Banxia	MOL003578	Cycloartenol	38.69	1.53	0.78
Banxia	MOL005030	gondoic acid	30.70	1.20	0.20
Banxia	MOL006936	10,13-eicosadienoic	39.99	1.22	0.20
Banxia	MOL006937	12,13-epoxy-9-hydroxynonadeca-7,10-dienoic acid	42.15	0.18	0.24
Banxia	MOL006944	8-Octadecenoic acid	33.13	1.15	0.14
Banxia	MOL006956	cyclo-(leu-tyr)	111.16	0.16	0.15
Banxia	MOL006957	(3S,6S)-3-(benzyl)-6-(4-hydroxybenzyl)piperazine-2,5-quinone	46.89	0.41	0.27
Zhishi	MOL000006	luteolin	36.16	0.19	0.25
Zhishi	MOL000131	EIC	41.90	1.16	0.14
Zhishi	MOL001798	neohesperidin_qt	71.17	0.26	0.27
Zhishi	MOL001803	Sinensetin	50.56	1.12	0.45
Zhishi	MOL001941	Ammidin	34.55	1.13	0.22
Zhishi	MOL002914	Eriodyctiol (flavanone)	41.35	0.05	0.24
Zhishi	MOL004328	naringenin	59.29	0.28	0.21
Zhishi	MOL005100	5,7-dihydroxy-2-(3-hydroxy-4-methoxyphenyl)chroman-4-one	47.74	0.28	0.27
Zhishi	MOL005828	nobiletin	61.67	1.05	0.52
Zhishi	MOL005849	didymin	38.55	0.60	0.24
Zhishi	MOL007879	Tetramethoxyluteolin	43.68	0.96	0.37
Zhishi	MOL009053	4-[(2S,3R)-5-[(E)-3-hydroxyprop-1-enyl]-7-methoxy-3-methylol-2,3-dihydrobenzofuran-2-yl]-2-methoxy-phenol	50.76	0.03	0.39
Zhishi	MOL013277	Isosinensetin	51.15	1.16	0.44
Zhishi	MOL013279	5,7,4'-Trimethylapigenin	39.83	1.01	0.30
Zhishi	MOL013352	Obacunone	43.29	0.01	0.77
Zhishi	MOL013430	Prangenin	43.60	0.80	0.29
Zhishi	MOL013433	prangenin hydrate	72.63	0.14	0.29
Zhishi	MOL013435	poncimarin	63.62	0.66	0.35
Zhishi	MOL013436	isoponcimarin	63.28	0.50	0.31
Zhishi	MOL013437	6-Methoxy aurapten	31.24	1.01	0.30
Zhishi	MOL013443	isolimonic acid	48.86	0.43	0.18
Zhishi	MOL013445	naringenin-4'-glucoside-7-rutinoside_qt	30.61	0.33	0.16
Gancao	MOL000098	quercetin	46.43	0.05	0.28
Gancao	MOL000211	Mairin	55.38	0.73	0.78
Gancao	MOL000239	Jaranol	50.83	0.61	0.29
Gancao	MOL000354	isorhamnetin	49.60	0.31	0.31
Gancao	MOL000359	sitosterol	36.91	1.32	0.75
Gancao	MOL000392	formononetin	69.67	0.78	0.21
Gancao	MOL000417	Calycosin	47.75	0.52	0.24
Gancao	MOL000422	kaempferol	41.88	0.26	0.24
Gancao	MOL000497	licochalcone a	40.79	0.82	0.29
Gancao	MOL000500	Vestitol	74.66	0.86	0.21
Gancao	MOL001484	Inermine	75.18	0.89	0.54
Gancao	MOL001789	isoliquiritigenin	85.32	0.44	0.15
Gancao	MOL001792	DFV	32.76	0.51	0.18
Gancao	MOL002311	Glycyrol	90.78	0.71	0.67
Gancao	MOL002565	Medicarpin	49.22	1.00	0.34
Gancao	MOL002844	Pinocembrin	64.72	0.61	0.18
Gancao	MOL003656	Lupiwighteone	51.64	0.68	0.37
Gancao	MOL003896	7-Methoxy-2-methyl isoflavone	42.56	1.16	0.20
Gancao	MOL004328	naringenin	59.29	0.28	0.21
Gancao	MOL004805	(2S)-2-[4-hydroxy-3-(3-methylbut-2-enyl)phenyl]-8,8-dimethyl-2,3-dihydropyrano[2,3-f]chromen-4-one	31.79	1.00	0.72
Gancao	MOL004806	euchrenone	30.29	1.09	0.57
Gancao	MOL004808	glyasperin B	65.22	0.47	0.44
Gancao	MOL004810	glyasperin F	75.84	0.43	0.54
Gancao	MOL004811	Glyasperin C	45.56	0.71	0.40
Gancao	MOL004814	Isotrifoliol	31.94	0.53	0.42
Gancao	MOL004815	(E)-1-(2,4-dihydroxyphenyl)-3-(2,2-dimethylchromen-6-yl)prop-2-en-1-one	39.62	0.66	0.35
Gancao	MOL004820	kanzonols W	50.48	0.63	0.52
Gancao	MOL004824	(2S)-6-(2,4-dihydroxyphenyl)-2-(2-hydroxypropan-2-yl)-4-methoxy-2,3-dihydrofuro[3,2-g]chromen-7-one	60.25	0.00	0.63
Gancao	MOL004827	Semilicoisoflavone B	48.78	0.45	0.55
Gancao	MOL004828	Glepidotin A	44.72	0.79	0.35
Gancao	MOL004829	Glepidotin B	64.46	0.46	0.34
Gancao	MOL004833	Phaseolinisoflavan	32.01	1.01	0.45
Gancao	MOL004835	Glypallichalcone	61.60	0.76	0.19
Gancao	MOL004836	echinatin	66.58	0.38	0.17
Gancao	MOL004838	8-(6-hydroxy-2-benzofuranyl)-2,2-dimethyl-5-chromenol	58.44	1.00	0.38
Gancao	MOL004841	Licochalcone B	76.76	0.47	0.19
Gancao	MOL004848	licochalcone G	49.25	0.64	0.32
Gancao	MOL004849	3-(2,4-dihydroxyphenyl)-8-(1,1-dimethylprop-2-enyl)-7-hydroxy-5-methoxy-coumarin	59.62	0.40	0.43
Gancao	MOL004855	Licoricone	63.58	0.53	0.47
Gancao	MOL004856	Gancaonin A	51.08	0.80	0.40
Gancao	MOL004857	Gancaonin B	48.79	0.58	0.45
Gancao	MOL004863	3-(3,4-dihydroxyphenyl)-5,7-dihydroxy-8-(3-methylbut-2-enyl)chromone	66.37	0.52	0.41
Gancao	MOL004864	5,7-dihydroxy-3-(4-methoxyphenyl)-8-(3-methylbut-2-enyl)chromone	30.49	0.90	0.41
Gancao	MOL004866	2-(3,4-dihydroxyphenyl)-5,7-dihydroxy-6-(3-methylbut-2-enyl)chromone	44.15	0.48	0.41
Gancao	MOL004879	Glycyrin	52.61	0.59	0.47
Gancao	MOL004882	Licocoumarone	33.21	0.84	0.36
Gancao	MOL004883	Licoisoflavone	41.61	0.37	0.42
Gancao	MOL004884	Licoisoflavone B	38.93	0.46	0.55
Gancao	MOL004885	licoisoflavanone	52.47	0.39	0.54
Gancao	MOL004891	shinpterocarpin	80.30	1.10	0.73
Gancao	MOL004898	(E)-3-[3,4-dihydroxy-5-(3-methylbut-2-enyl)phenyl]-1-(2,4-dihydroxyphenyl)prop-2-en-1-one	46.27	0.41	0.31
Gancao	MOL004904	licopyranocoumarin	80.36	0.13	0.65
Gancao	MOL004905	3,22-Dihydroxy-11-oxo-delta(12)-oleanene-27-alpha-methoxycarbonyl-29-oic acid	34.32	-0.06	0.55
Gancao	MOL004907	Glyzaglabrin	61.07	0.34	0.35
Gancao	MOL004908	Glabridin	53.25	0.97	0.47
Gancao	MOL004910	Glabranin	52.90	0.97	0.31
Gancao	MOL004911	Glabrene	46.27	0.99	0.44
Gancao	MOL004912	Glabrone	52.51	0.59	0.50
Gancao	MOL004913	1,3-dihydroxy-9-methoxy-6-benzofurano[3,2-c]chromenone	48.14	0.48	0.43
Gancao	MOL004914	1,3-dihydroxy-8,9-dimethoxy-6-benzofurano[3,2-c]chromenone	62.90	0.40	0.53
Gancao	MOL004915	Eurycarpin A	43.28	0.43	0.37
Gancao	MOL004935	Sigmoidin-B	34.88	0.42	0.41
Gancao	MOL004941	(2R)-7-hydroxy-2-(4-hydroxyphenyl)chroman-4-one	71.12	0.41	0.18
Gancao	MOL004945	(2S)-7-hydroxy-2-(4-hydroxyphenyl)-8-(3-methylbut-2-enyl)chroman-4-one	36.57	0.72	0.32
Gancao	MOL004948	Isoglycyrol	44.70	0.91	0.84
Gancao	MOL004949	Isolicoflavonol	45.17	0.54	0.42
Gancao	MOL004957	HMO	38.37	0.79	0.21
Gancao	MOL004959	1-Methoxyphaseollidin	69.98	1.01	0.64
Gancao	MOL004961	Quercetin der	46.45	0.39	0.33
Gancao	MOL004966	3'-Hydroxy-4'-O-Methylglabridin	43.71	1.00	0.57
Gancao	MOL004974	3'-Methoxyglabridin	46.16	0.94	0.57
Gancao	MOL004978	2-[(3R)-8,8-dimethyl-3,4-dihydro-2H-pyrano[6,5-f]chromen-3-yl]-5-methoxyphenol	36.21	1.12	0.52
Gancao	MOL004980	Inflacoumarin A	39.71	0.73	0.33
Gancao	MOL004985	icos-5-enoic acid	30.70	1.22	0.20
Gancao	MOL004988	Kanzonol F	32.47	1.18	0.89
Gancao	MOL004989	6-prenylated eriodictyol	39.22	0.40	0.41
Gancao	MOL004990	7,2',4'-trihydroxy-5-methoxy-3-arylcoumarin	83.71	0.24	0.27
Gancao	MOL004991	7-Acetoxy-2-methylisoflavone	38.92	0.74	0.26
Gancao	MOL004993	8-prenylated eriodictyol	53.79	0.43	0.40
Gancao	MOL004996	gadelaidic acid	30.70	1.20	0.20
Gancao	MOL005000	Gancaonin G	60.44	0.78	0.39
Gancao	MOL005001	Gancaonin H	50.10	0.60	0.78
Gancao	MOL005003	Licoagrocarpin	58.81	1.23	0.58
Gancao	MOL005007	Glyasperins M	72.67	0.49	0.59
Gancao	MOL005008	Glycyrrhiza flavonol A	41.28	-0.09	0.60
Gancao	MOL005012	Licoagroisoflavone	57.28	0.71	0.49
Gancao	MOL005016	Odoratin	49.95	0.42	0.30
Gancao	MOL005017	Phaseol	78.77	0.76	0.58
Gancao	MOL005018	Xambioona	54.85	1.09	0.87
Gancao	MOL005020	dehydroglyasperins C	53.82	0.68	0.37
Shengjiang	MOL000358	beta-sitosterol	36.91	1.32	0.75
Shengjiang	MOL002467	6-gingerol	35.64	0.54	0.16
Shengjiang	MOL002495	6-shogaol	31.00	1.07	0.14
Shengjiang	MOL006129	6-methylgingediacetate2	48.73	0.55	0.32

### Construct the weighted gene regulatory network of ICH

**Fig. 2:**
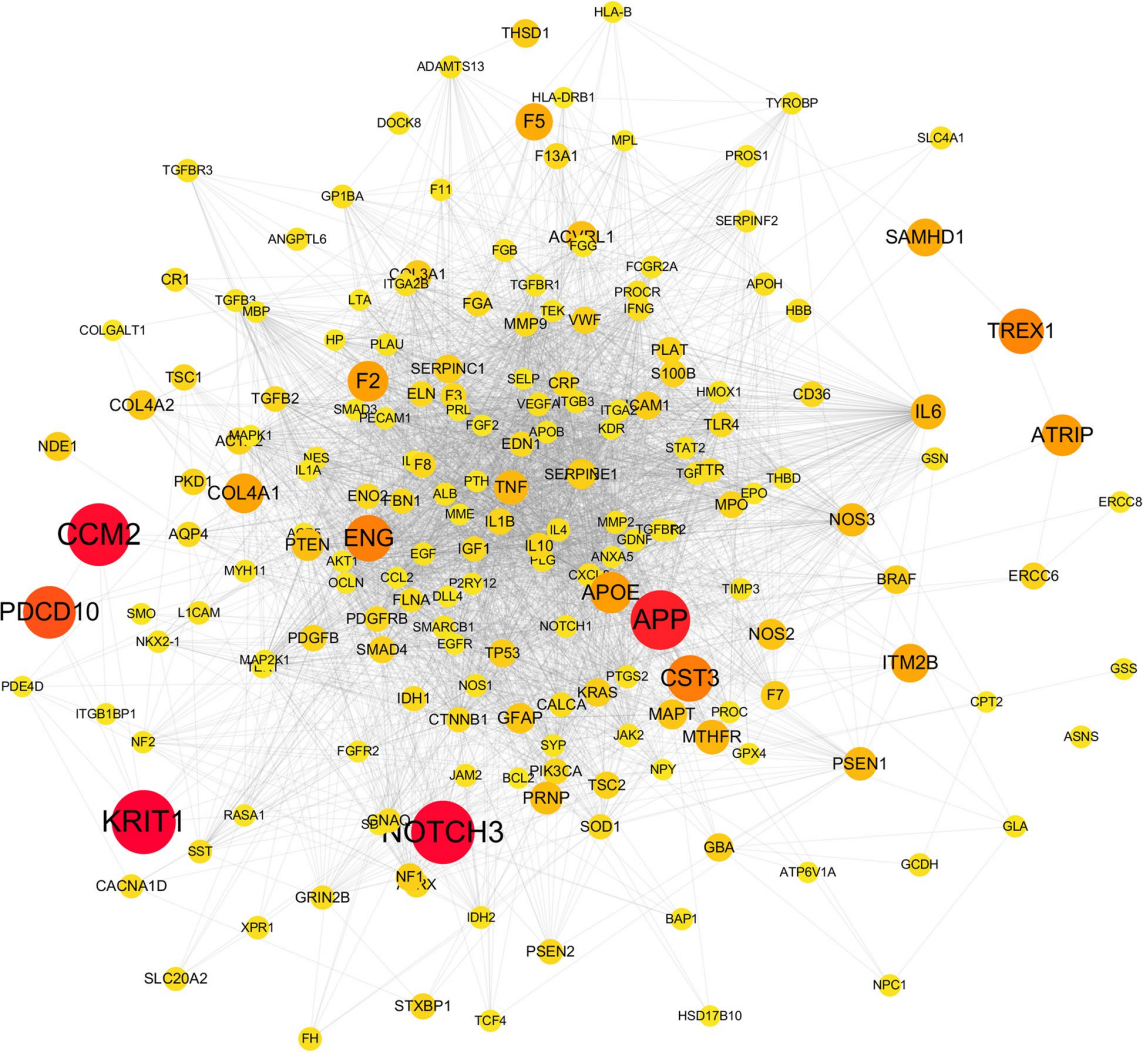
The disease weight gene regulatory network of ICH

Constructing and analyzing weighted gene regulatory network is the basis and key step to understand the pathogenesis of ICH and provide intervention strategies. We obtained the genes related to ICH from Genecard database. We selected 2648 genes larger than the average score as the genes related to ICH. At the same time, we used dispenet for comparison, and found that the selected genes could cover 88% of the dispenet library. The comprehensive PPI network, which combined by from several PPI databases were download from the CMGRN [30] and PTHGRN [31]. A total of 999 genes were extracted from DisGeNET and OMIM related to ICH, and mapped to PPI network, and a weighted gene regulatory ne48.82twork was constructed. The weighted gene regulatory network contains 999 nodes and 46,432 edges (Fig. 2). In the disease network, the degree indicates the importance of nodes. We further analyze it by using Network Analyzer software. In PPI network, the target average degree of different ingredients is 48.82. Among the top weighted targets, APP [32], NOTCH3 [33], KRIT1, CCM2, PDCD10, CST3, ENG, TREX1, ATRIP, APOE, etc.

### Common ingredients of Chinese herbal medicines in DTT

It can be seen from Table 3 that there are 12 active ingredients shared by more than two kinds of herbs in DTT. For example, β-sitosterol is a common ingredient of ginseng, Arisaema cum, orange, ginger and Pinellia ternate. Studies have found that β-sitosterol has a strong inhibitory effect on oxygen free radicals and a strong antioxidant effect on oils and fats [34]. Naringin is shared by Fructus Aurantii Immaturus, Glycyrrhrizae Radix and Exocarpium Citri Grandis. Naringin can obviously alleviate permanent nerve injury and has protective effect. Its mechanism is to down-regulate the expression of NOD2, RIP2 and MMP-9, up-regulate the expression of claudin-6 and protect the blood–brain barrier [35]. 6-gingerol is a common ingredient of Pinellia ternata and Ginger. 6- gingerol may protect PC12 cells against apoptosis induced by Aβ 1–42 through PI3K/Akt/GSK-3β signaling pathway, and has protective effect on nerve cells [36]. In addition, linolenic acid is shared by Pinellia ternata and Arisaema cum laude. Experiments show that increasing DHA level in brain can limit the expression of inflammatory factors after TBI and accelerate the recovery of nerve function [37].

**Table 3:** 12 active ingredients shared by more than two kinds of herbs in DTT

ingredients	Herbs
6-shogaol	*Pinellia ternata* (Thunb.) Makino; *Zingiber officinale* Roscoe
β-sitosterol	*Pinellia ternata* (Thunb.) Makino; *Zingiber officinale* Roscoe; *Citrus reticulata* Blanco; *Panax ginseng* C.A.Mey
didymin	*Citrus acida* Pers; *Citrus reticulata* Blanco
EIC	*Pinellia ternata* (Thunb.) Makino; *Citrus reticulata* Blanco; *Arisaema heterophyllum* Blume
kaempferol	*Glycyrrhiza uralensis* Fisch. ex DC.; *Panax ginseng* C.A.Mey
linolenic acid	*Pinellia ternata* (Thunb.) Makino; *Arisaema heterophyllum* Blume
methyl linoleate	*Arisaema heterophyllum* Blume; *Panax ginseng* C.A.Mey.(Renshen)
naringenin	*Citrus reticulata* Blanco; *Citrus reticulata* Blanco (Juhong); *Glycyrrhiza uralensis* Fisch. ex DC
neohesperidin_qt	*Citrus acida* Pers; *Citrus reticulata* Blanco
Obacunone	*Citrus acida* Pers; *Citrus reticulata* Blanco
sitosterol	*Arisaema heterophyllum* Blume; *Glycyrrhiza uralensis* Fisch. ex DC
Stigmasterol	*Pinellia ternata* (Thunb.) Makino; *Arisaema heterophyllum* Blume; *Panax ginseng* C.A.Mey

### Special ingredients of Chinese herbal medicine in DTT

Apart from the common ingredients, most herbs have their specific ingredients. For example, the main ingredient of ginseng is ginsenosides. Studies by Shi et al. and others have found that neural stem cells are activated and proliferated after intracerebral hemorrhage [38]. Ginsenosides can induce the proliferation of neural stem cells and improve motor function after intracerebral hemorrhage. Ferulic acid is one of the main ingredients of Arisaema cum laude. Ferulic acid reduces the expression of phosphorylated IKK and the transport of NRF2 and NF-kappab to the nucleus, thus inhibiting the activities of IL-6 and NF-kappab promoters. These data indicate that ferulic acid could inhibit inflammatory by mediating IKK/NF-kappab signaling pathway [39]. Naringin (DTT187) is one of the most effective ingredients in tangerine peel. Liu Wei et al. found that naringin pre-intervention can effectively alleviate cerebral ischemia–reperfusion injury, and the protective effect is closely related to the decrease of Cx43 expression in astrocytes [40].

### Target prediction of active ingredients

In order to deduce the underlying mechanism of DTT in the treatment of ICH, 181 active ingredients and 1544 target points were employed to construct the ingredient target network. Among them, several active ingredients are associated with multiple targets, resulting in 41,650 target relationships between 181 active ingredients and 1544 targets. The average target number for each ingredient is 47.11. The results indicate that DTT is a multi-ingredient and multi-target therapy for ICH. Among these ingredients, the target of MOL000131 (degree = 333) is the most, followed by MOL000358 (degree = 300), MOL002495 (degree = 246), MOL013156 (degree = 217), MOL000442 (degree = 156) and MOL000432 (degree = 146). Most of these ingredients are related to inflammation and oxidation related pathways of DTT. For example, 6-gingerol has anti-inflammatory and antioxidant effects, which can improve the activities of SOD), glutathione peroxidase and catalase in organisms, eliminate hydroxyl radicals and superoxide radicals, and reduce lipid peroxides in tissues [41]. Stigmasterol can significantly inhibit the increase of ROS induced by Ang ii in A7r5 cells, and increase the activities of SOD and CAT enzymes in A7r5 cells treated by Ang ii. In addition, stigmasterol can obviously inhibit the increased iNOS mRNA and protein levels of COX-2 and iNOS induced by LPS, and has anti-inflammatory effect [42]. The role of other ingredients in the treatment of ICH has been described in the chapters of "Shared Ingredients of Chinese Herbal Medicine in DTT" and "Specific Ingredients of Chinese Herbal Medicine in DTT". These results proved the important role of these ingredients in ICH and further suggested that the multi-ingredient role of DTT in treating ICH.

In the ingredient target network, the average target degree of different targets is 3.86. Among the 20 weighted targets, ABCG2, ABCB1, ALOX5, ALOX15, ALOX12, etc. Interestingly, most of these targets are related to oxidation and inflammation, which have been confirmed to be related to the pathogenesis of ICH, and may indicate the potential therapeutic mechanism of DTT on ICH. For example, caspase-3 is closely related to cerebral hemorrhage and is one of the main factors of neuronal apoptosis. The activation of caspase-3 after intracerebral hemorrhage may be the mechanism of ischemia–reperfusion injury caused by secondary cerebral ischemia around hematoma [43]. In addition, the early release of cytochrome C may be related to hematoma occupation, ischemia and hypoxia of peripheral neurons caused by brain edema, energy metabolism disorder, calcium overload, oxidative stress and the production of a large number of free radicals.The mechanism by which DTT can block the release of cytochrome C is related to the mitochondrial permeability transition pore channel and the Bcl-2 family of proteins. By blocking the release of cytochrome C into the cytoplasm, the apoptosis of neurons was prevented [44].

These results suggest that DTT can treat ICH synergistically by regulating inflammation and antioxidant function, which further confirms the multi-target role of DTT in ICH treatment.

### Functional proteins selection and validation based on dtt potential effect space

**Fig. 3:**
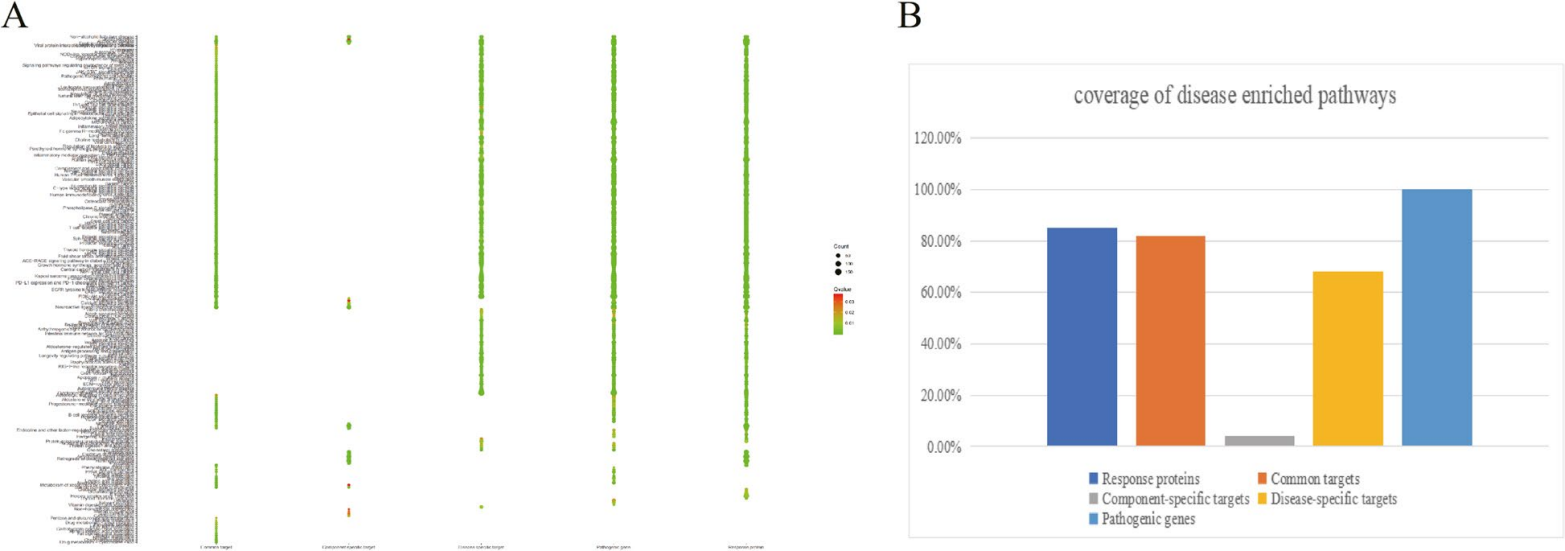
Functional proteins validation. KEGG pathways distribution of response proteins, common targets, ingredient-specific targets, disease-specific targets and pathogenic genes (**A**); the coverage rate of response proteins, common targets, ingredient-specific targets, and disease-specific targets enriched pathways compared with pathogenic gene-enriched pathways of ICH (**B**)

We constructed a C-T-P network based on PPI using the weighted pathogenic gene regulatory network of ICH and the target network of active ingredients. In this network, it contains 2786 nodes and 45,647 edges. We extracted the relationship between drug targets and pathogenic genes from C-T-P network and constructed DTT potential effect space. Node importance is an important factor to be considered in optimizing network. At present, the methods to describe node importance mainly include degree, betweenness, closeness to centrality, shortest path and so on. These methods mainly depend on the nature of a certain aspect of the network to describe the importance of nodes in the network. In this study, we designed a new network importance calculation method, which takes into account the influence and connectivity of nodes. The comparison results show that the therapeutic response protein obtained by our node importance calculation method accounts for 92.57% of the pathogenic gene enrichment pathway in go function enrichment analysis, which shows that our method has good accuracy. There are three types of proteins in DTT potential effect space. The first is the direct interaction between pathogenic genes and drug targets. We define this category as the basic common targets. The second category is the interaction of disease-specific targets. The third category is the interaction of ingredient-specific targets. In order to determine whether the therapeutic response protein we selected from DTT PES is optimal, we examined the coverage of the enrichment pathway of therapeutic response protein, common target, ingredient-specific target and disease-specific target in the enrichment pathway of pathogenic genes from the functional level. The results showed that the coverage rate of therapeutic response protein was as high as 84.9%, which was 3.1%, 80.6% and 16.8% higher than the three, indicating that the therapeutic response protein we selected had better functional coverage (Fig. 3A and B).

**Fig. 4:**
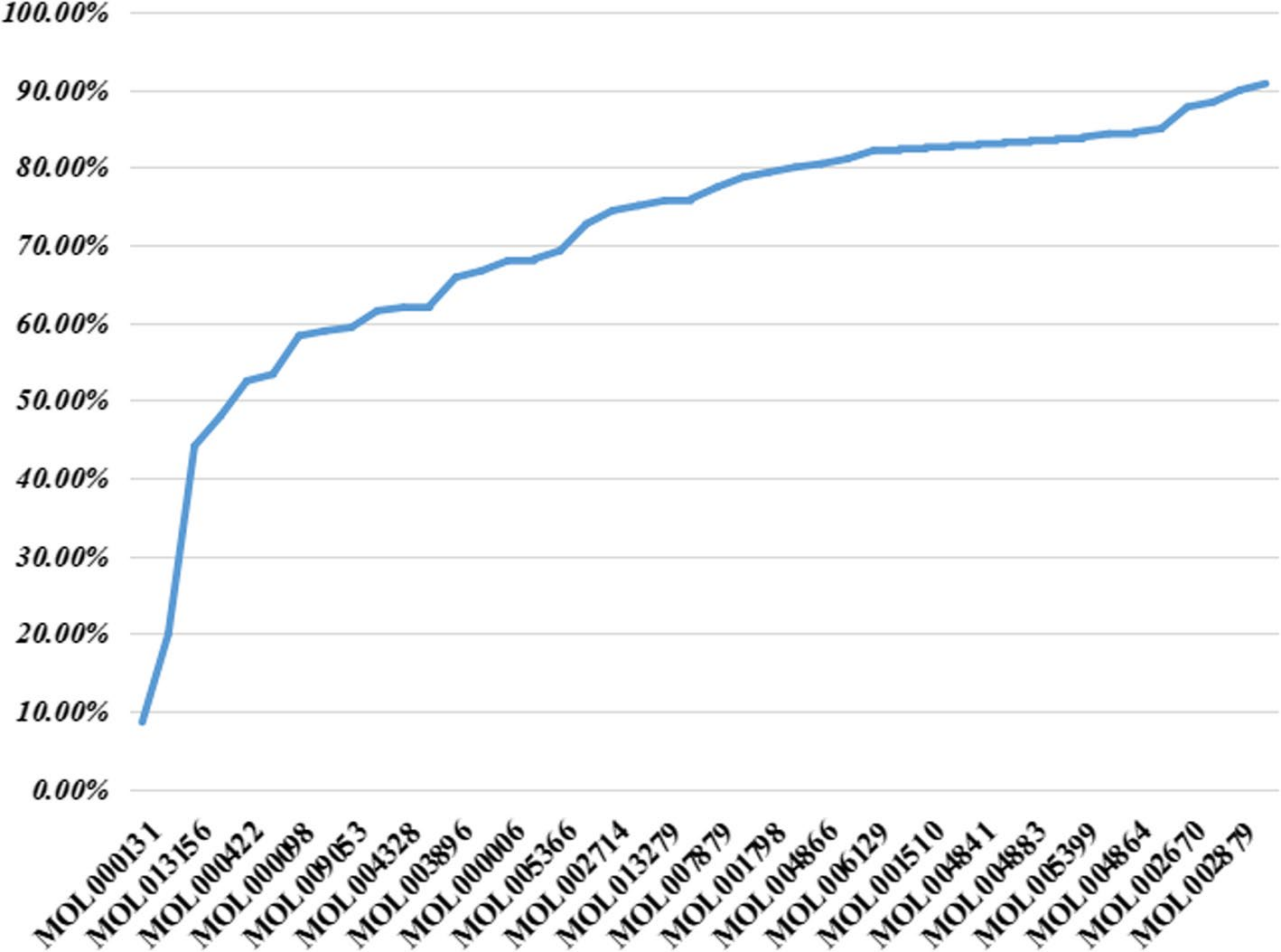
The contribution accumulation rate of DTT FCIG

The contribution coefficient model is constructed to optimize the effective ingredients and obtain FCIG values, which can be used to clarify the potential mechanism of DTT in treating ICH. According to the result of contribution accumulation rate (Fig. 4), the cumulative contribution rate of the first 44 ingredients reaches 90.75%, which is selected as FCIG. Include EIC (MOL000131), 6-shogaol (MOL002495), [(2r)-2-[[(2r)-2-(benzoylamino)-3-phenylpropyl] amino] methyl]-3-phenylpropyl] acetate (*n* = 1) Beta-sitosterol (MOL000358), kaempferol (MOL000422), methyl linoleate (MOL001641), etc.

**Fig. 5:**
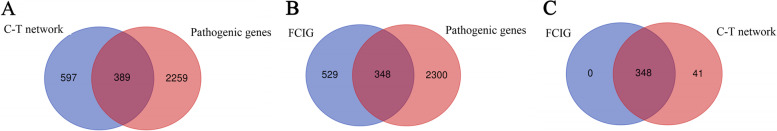
Venn diagram was used to visualize the overlap number of pathogenic genes between C-T network targets (**A**) and FCIG network targets (**B**), and the overlap number of C-T network targets overlap pathogenic genes and FCIG network targets overlap pathogenic genes

There is a high consistency between FCIG and C-T network in the number of pathogenic genes. In order to evaluate whether the genes in FCIG are related to ICH, we compared the coverage of pathogenic genes in FCIG and C-T networks. We collected the published literature and the known ICH pathogenic genes in the database, which were verified by the average score of GeneCards greater than 6. It was found that there were 389 pathogenic genes in C-T network and 348 pathogenic genes in FCIG of DTT (Fig. 5A and B). The FCIG of DTT reached 89.5% of C-T network (Fig. 5C). This result shows that our FCIG detection model can maximize the coincidence degree of pathogenic genes in the formula CTT network.

**Fig. 6:**
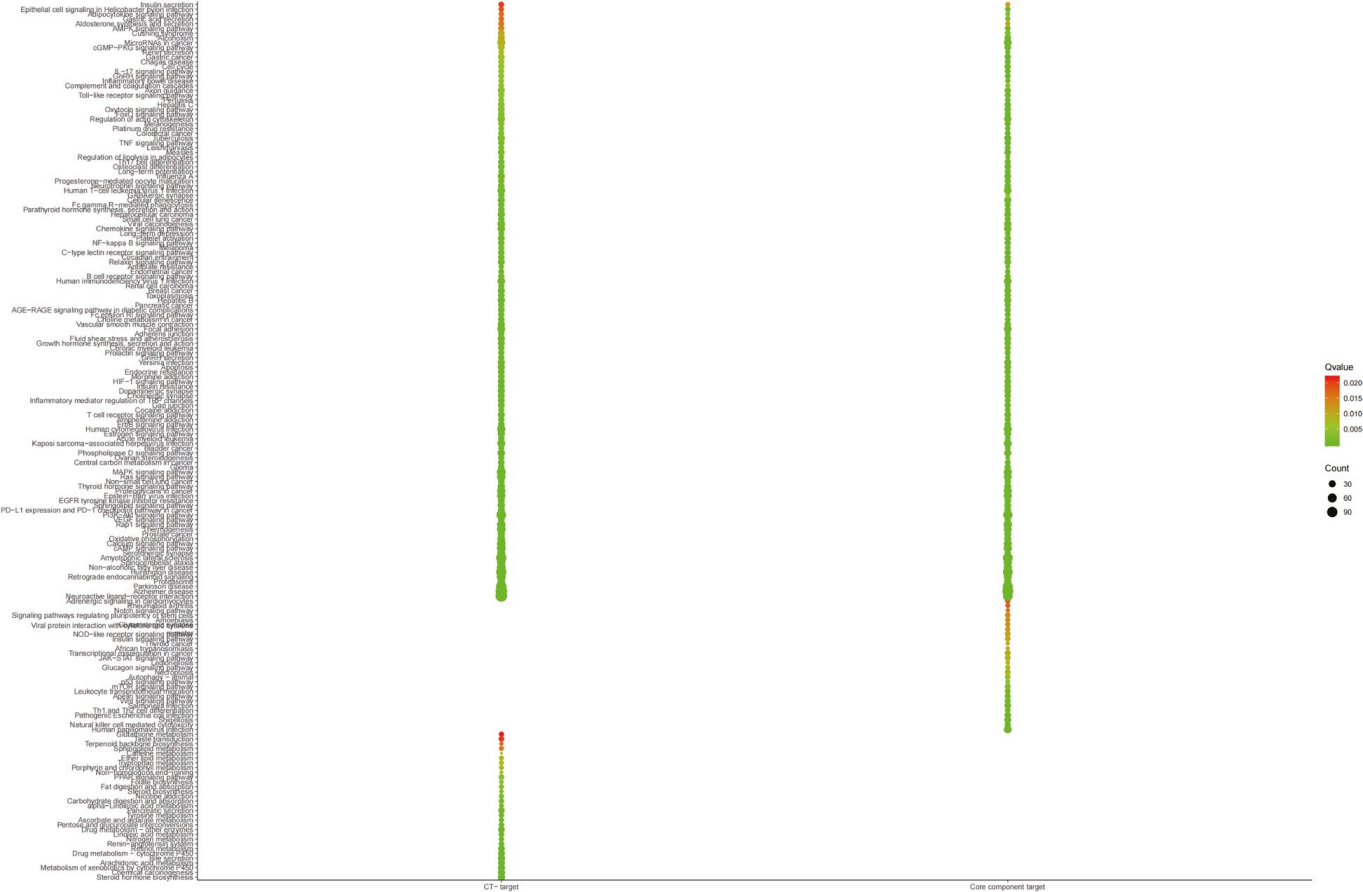
The functional similarity analysis between targets of C-T network and FCIG

FCIG is highly consistent with C-T network in gene function level: another index to evaluate the importance of FCIG network is determined by their functional consistency, which can be evaluated by their enrichment pathway in KEGG. The purpose is to test whether FCIG found in DTT can represent its complete C-T network. This result shows that our FCIG detection model can cover C-T network functions to the maximum extent (Fig. 6). There is a high degree of coincidence between FCIG and C-T network in topology: node degree is a key topological parameter to characterize network topology, and it is also the most influential node in the network, so we use it to further determine the importance of FCIG. A mathematical model was established to evaluate the importance of network FCIG in DTT. Then the CC value of FCIG in DTT was calculated by topological parameters. The results show that FCIG can cover 80.44% of C-T composed of DTT. The results show that the FCIG in DTT is consistent with the pathway and pathogenic genes, and our FCIG model can maximize the coverage of network topology and C-T network composed of DTT.

### Functional core ingredients selection and validation

**Fig. 7:**
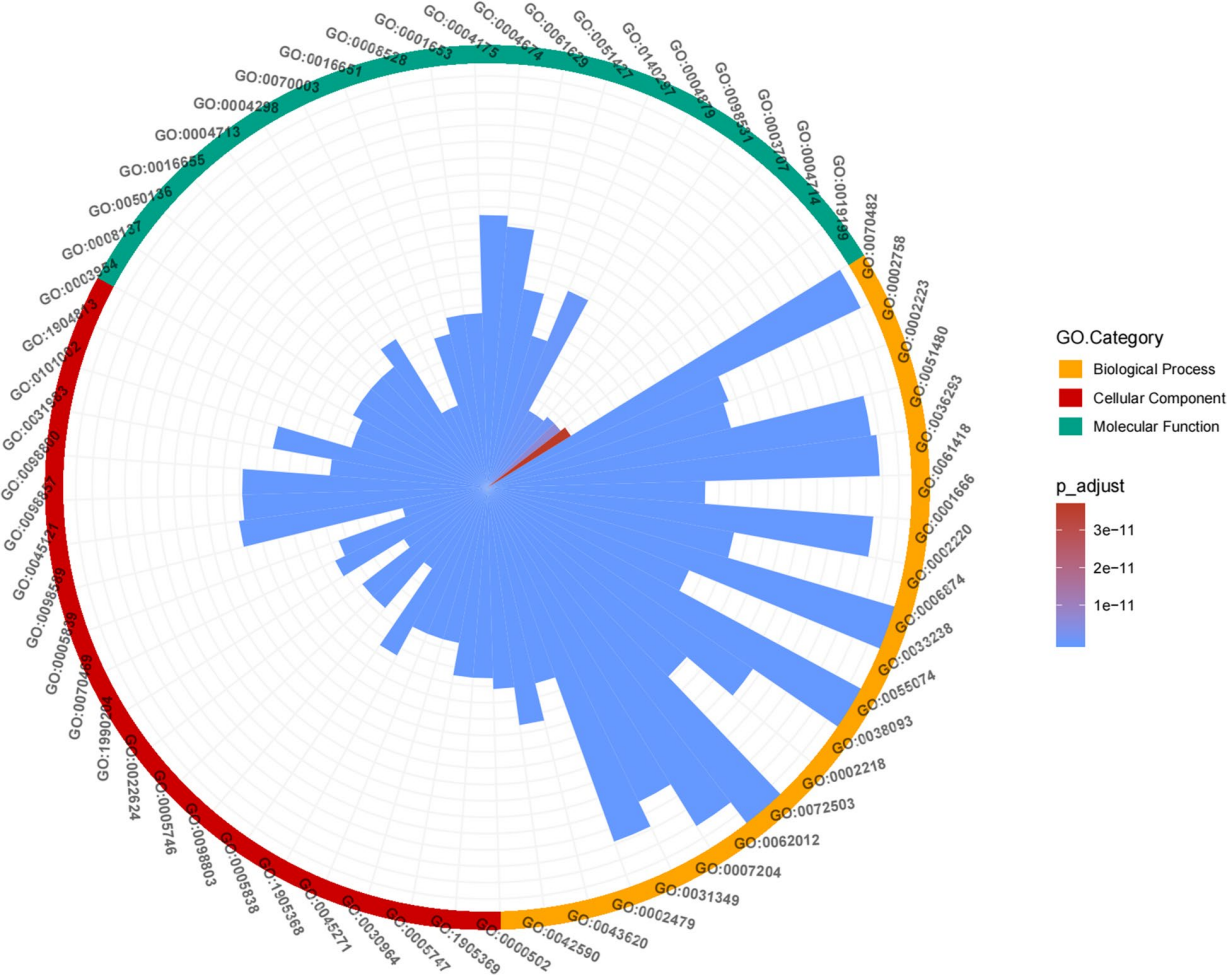
Go enrichment analysis of the targets of FCIG

GO enrichment analysis was performed using the R software clusterProfiler package to identify the biological functions of the main targets with p values < 0.05. To further profile the combined effects of DTT, all targets that interacted with FCIG in DTT were enriched by GO enrichment analysis (Fig. 7).

**Fig. 8:**
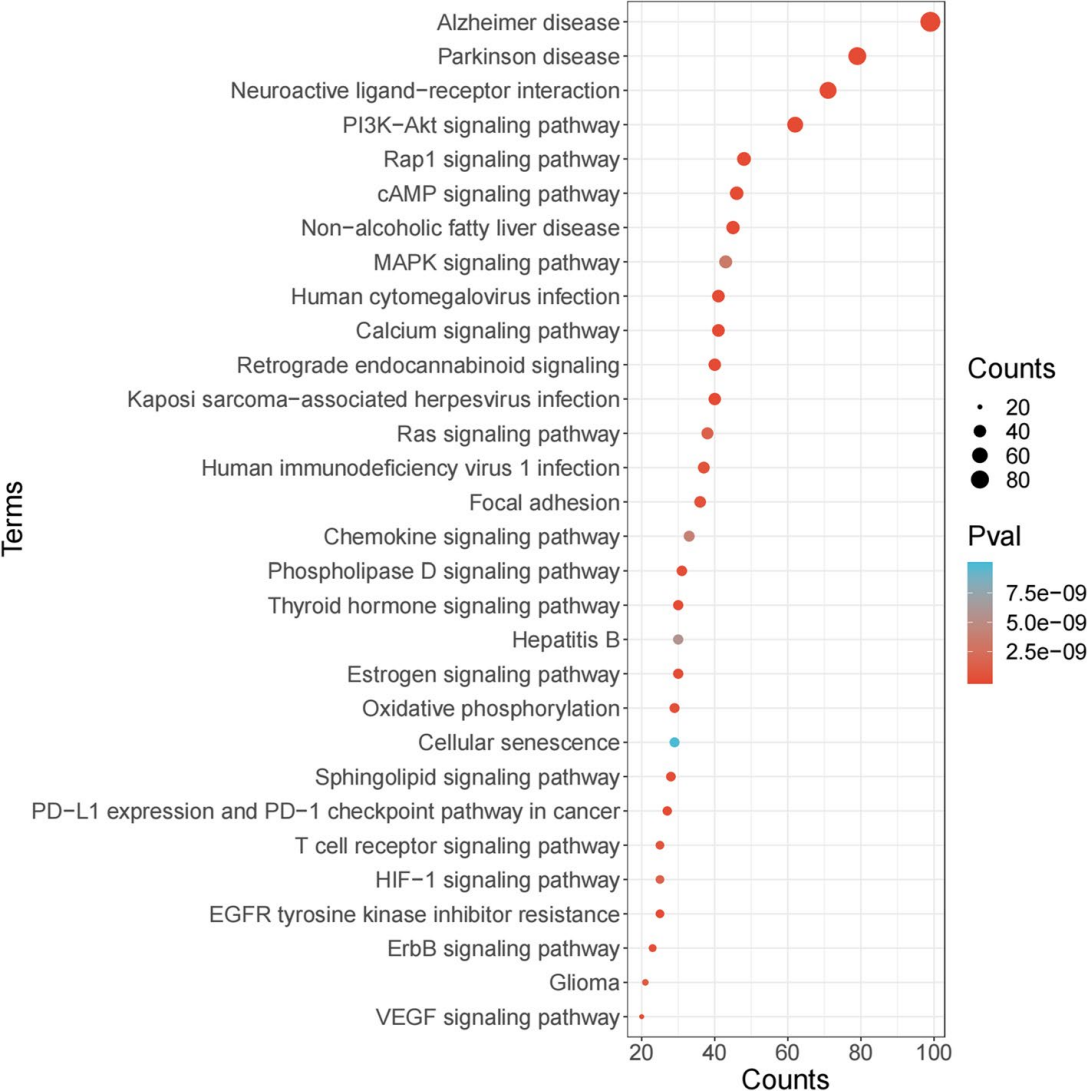
Pathway enrichment analysis of the targets of DTT

DTT regulatory targets were abundantly expressed in biological processes related to inflammatory response, according to GO analysis.For example, reducing oxidative stress (GO:0,070,482, GO:0,036,293, GO:0,061,418) leukocyte activation involved in inflammatory responses (GO:0,002,758), the production of molecular mediators involved in inflammatory responses (GO:0,070,498), and inflammatory responses to antigen stimulation (GO:0,002,220). These results confirmed that DTT could treat ICH by reducing inflammatory reaction, reducing oxidative stress and inhibiting apoptosis (Fig. 8).

### Potential mechanisms analysis of DTT in treating ICH

**Fig. 9:**
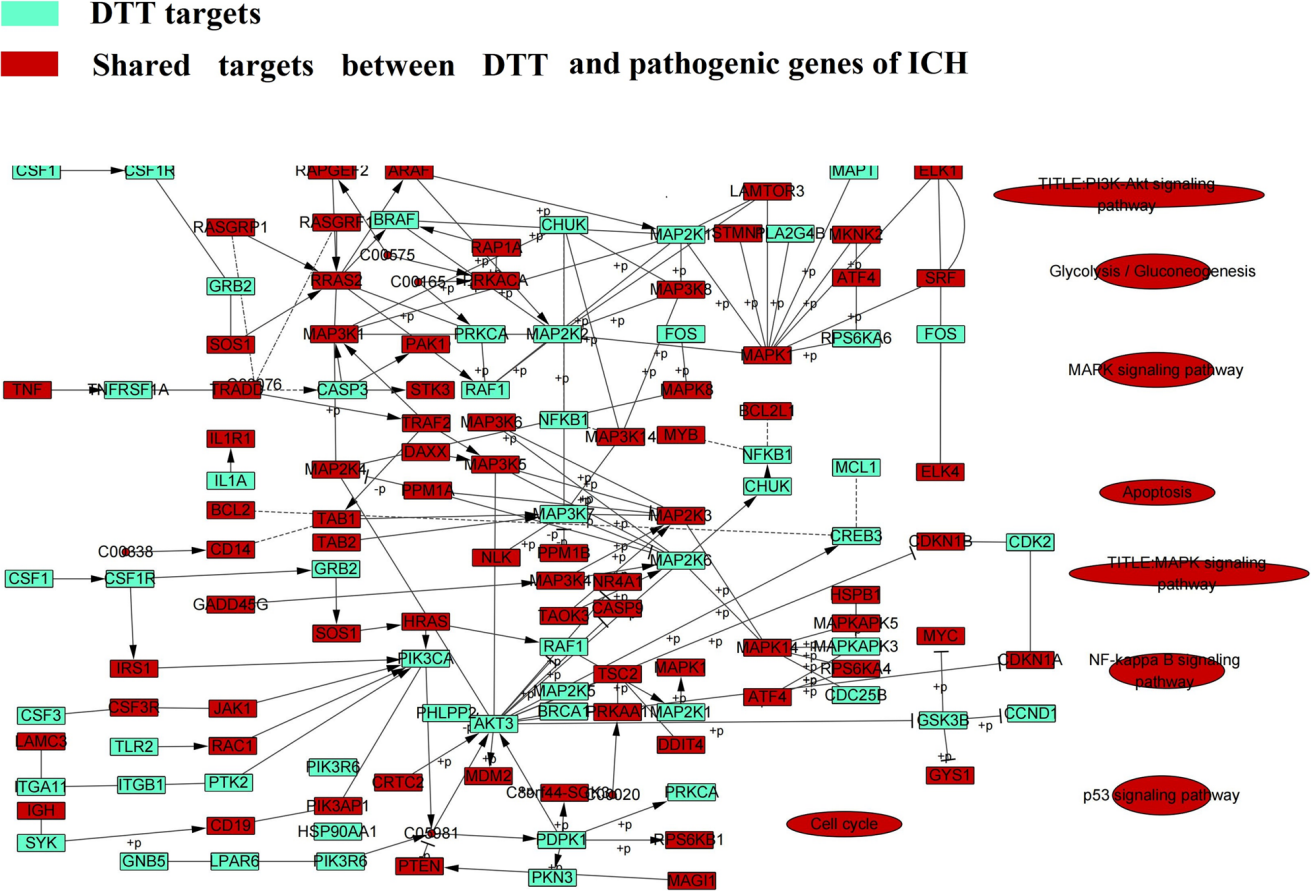
Distribution of ingredients of DTT on the compressed ICH pathway

Pathological changes of ICH are related to many factors, among which oxidative stress and apoptosis are the key factors. When oxidative stress occurs, a large amount of reactive oxygen species will damage the blood–brain barrier, mediate demyelination and axonal injury, and activate various signal pathways to induce and aggravate autoimmune inflammation and neuronal apoptosis. We found 100 pathways shared by pathogenic genes, including MAPK signaling pathway (hsa04010), PI3K-Akt signaling pathway (HSA 04,151) and so on. More and more evidences show that these pathways and the pathogenesis or therapeutic targets of ICH, such as MAPK signaling pathway (hsa04010), mainly regulate protein synthesis, cell proliferation and apoptosis. MAPK plays a positive role in the initiation and recognition of oxidative stress, which has a positive correlation with brain injury. Using MAPK-related protein blockers can play a role in brain protection, and the effect of combining blockers is better. This suggests that multiple blocking of oxidative stress-related signaling pathways is one of the research ideas to reduce brain injury. DTT can play a role in pathological changes of cerebral hemorrhage by regulating apoptosis and oxidative stress pathway. In order to deduce the underlying mechanism of DTT on ICH, a comprehensive signal pathway was constructed at the system level. In order to figure out the location of DTT target on the pathway, the first three columns were defined as the upstream and the others were defined as the downstream of the pathway. We found that PI3K-Akt signaling pathway (hsa04151) is one of the most important pathways in the treatment of ICH with DTT (Fig. 9).

### Experimental validation in vitro

**Fig. 10:**
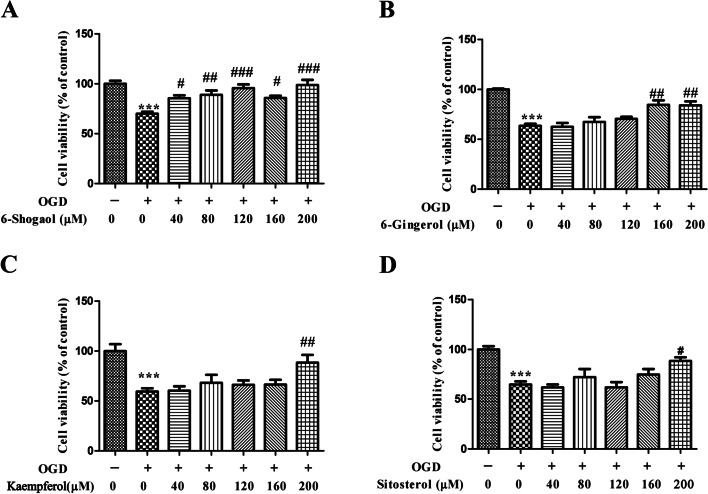
Effects of 6-Shogaol (**A**),6-Singerol (**B**), kaempferol (**C**), and sitostrol (**D**) on cell viabilities. ***p < 0.001 compared with control group. #*p* < 0.05, #*p* < 0.05, ###*p* < 0.001 compared with the OGD group

The effects of 6-Shogaol, 6-Singerol, kaempferol, and sitostrol on OGD model were evaluated. Compared with control group, the cell viability of OGD groups were significantly reduced. Compared with control group, the effect of cell viability was significantly decreased by 31.47% in the hypoxia treated cells (Fig. 10). However,6-Singerol (40 μM、80 μM、120 μM、160 μM and 200 μM) markedly increased the cell viability level by18.81%、19.98%、21.96%、16.53% and 23.47%.Compared with control group, the effect of cell viability was significantly decreased by 36.40% in the hypoxia treated cells. However,6-Singerol (160 μM and 200 μM) markedly increased the cell viability level by 20.96% and 20.44%.Compared with control group, the effect of cell viability was significantly decreased by 40.61% in the hypoxia treated cells. However, kaempferol (200 μM) markedly increased the cell viability level by 29.01%.Compared with control group, the effect of cell viability was significantly decreased by 35.08% in the hypoxia treated cells. However, sitostrol (200 μM) markedly increased the cell viability level by 23.65%.The above results demonstrated that 6-Shogaol, 6-Singerol, kaempferol, and sitostrol possessed protect effect in hypoxia treated HT22 cells.

## Discussion

Reducing non-pharmacological ingredients and improving curative effects are the main objectives of formula optimization. According to the theory of Chinese medicine, different herb medicines make up prescriptions, but whether the herbs or ingredients in prescriptions are necessary, especially for certain indications, needs to be analyzed and verified. By optimizing the formula, the medicinal materials or ingredients with certain efficacy were screened out, which made the formula more clarified and the efficacy more enhanced. In order to better capture the clinical efficacy of classical prescriptions, bioinformatics and systems pharmacology methods were combined to study the coverage rate of changed targets, and the relevant functional analysis was made on the coverage of pathogenic genes. The changed targets responds to the combination changes of different herbs and different chemical ingredients in each formula. In order to find the optimal FCIG, the optimization space definition and ingredient reverse search strategy were applied to evaluate it. At present, how to optimize and obtain FCIG and deduced underlying mechanisms is the foundation of TCM research. TCM analysis focus on the holistic view and regards the body as a whole. Systems pharmacology focuses on analyzing the action mechanisms of formula from a systematic and holistic perspective, which accords with the theoretical system of TCM research. Systems pharmacology emphasizes the multi-target regulation of multiple signal pathways to promote the synergistic effect of drugs and reduce toxic and side effects. At present, systems pharmacology has been widely used in the research of TCM formulas, especially to determine the molecular mechanism of treating complex diseases with TCM formula. However, there are few reports on optimization of TCM formulas based on systems pharmacology. On this basis, a comprehensive optimization strategy of DTT based on network pharmacology is proposed, and the key ingredients of DTT in treating ICH are obtained, and the potential mechanism of these ingredients is analyzed.

Our method has two advantages: 1. In the process of analyzing the treatment mechanism, network pharmacology has formed a fixed analysis rule, that is, according to the chemical properties of Chinese medicine ingredients, through screening, predicting targets and analyzing potential mechanisms of action. This flow chart solves the molecular mechanism of some prescriptions for treating complex diseases with TCM, but there is also a problem that there is no reference in the optimization space. In this paper, we identified the representative pathogenic genes of ICH, and these pathogenic genes have been reported in the literature. For example, APP, NOTCH3 weighted gene regulatory network of ICH, Zhu Bin et al. found that amyloid precursor protein (APP) mutation can cause typical pathological changes of AD and perivascular amyloid deposition. Fu Jiayu et al. showed that NOTCH3 gene mutation may cause cerebral hemorrhage by changing the structure and function of cerebral small vessels. We present a reverse optimization model based on the association between disease genes and ingredient targets is proposed, which provides space for optimization based on effective proteins. This method can well determine the optimization space of the target. Second, reverse searching related ingredients based on the optimized space provided by effective protein. The results showed that the enrichment functional pathway of effective protein could cover 96% of the enrichment functional pathway of disease genes. It is proved that our strategy of selecting effective proteins to construct the target optimization space is correct. On the basis of optimization space provided by effective protein, CI model was used to optimize the contribution degree, and finally 82kgec was optimized. The target of FCIG is closely related to the pathogenesis and functional annotation of ICH. This proves the reliability of our optimization space and CI model again.

At present, network pharmacology provides a powerful tool for exploring the compatibility and action mechanism of TCM prescriptions. However, there are some limitations. For example, more main active ingredients should be considered in animal medical research of DTT. We will verify the efficacy and mechanism of active ingredients in treating ICH through in vivo or in vitro experiments. The results show that the model has good accuracy in screening FCIG in TCM formula and provides reference for optimization and mechanism deduction of TCM formulas.

## Conclusion

A network pharmacology model-based bioinformatics algorithm was established to obtain functional core ingredients group and decode the mechanisms of DTT in the treatment of ICH. Compared with other published work, potential effect space construction strategy based on novel node importance calculation method and validation strategy and maximum targeting weight model for mechanism speculation were reported. In addition, this new systemic pharmacology model closely combines the representative pathogenic genes of ICH, which can reflect the incidence of ICH well, and can effectively screen out the core ingredients of DTT in treating ICH.

Results of the in vitro validation that 6-Shogaol, 6-Singerol, kaempferol, and sitostrol possessed protect effect in hypoxia treated HT22 cells demonstrated that the core ingredient group selected by us based on system pharmacology had significant effect on ICH.

Our research is a computational mining work based on pharmacological basic data, which provides a feasible scheme to reduce the verification scale for the experiment, provides methodological reference for optimization of core ingredients group and interpretation of the molecular mechanism in the treatment of complex diseases using TCM. Our model has been proved to be effective in the compound optimization of DTT for the treatment of ICH. In future studies, we hope to apply this model to more studies and get better improvements, so as to provide methodological reference for the treatment of ICH with Chinese medicine.

## Supplementary Information

**Additional file 1:** **Supplementary Table 1.** Protein-Protein Interaction Network.

## Data Availability

The datasets used or analysed during the current study are available from the corresponding author. Similarity Ensemble Approach: https://sea.bkslab.org/ GeneCards database: https://www.genecards.org/STRING: https://cn.string-db.org/ BioGRID: https://thebiogrid.org/ HPRD: http://www.hprd.org/.

## Abbreviations

TCM: Traditional Chinese medicine
ICH: Hypertensive cerebral hemorrhage
PES: Potential effect space
CI: Contribution index
DTT: Ditan decoction
FCIG: Functional core ingredients group
DL: Drug-likeness
GO: Gene ontology
PPI: Protein–protein interactions
